# Cardiovascular Outcomes in Patients With Diabetes and Chronic Liver Disease

**DOI:** 10.31083/RCM49626

**Published:** 2026-04-24

**Authors:** Alfredo Caturano

**Affiliations:** ^1^Department of Human Sciences and Promotion of the Quality of Life, San Raffaele Roma University, 00166 Rome, Italy

## 1. Introduction

Cardiovascular disease remains the leading cause of morbidity and mortality worldwide, accounting for a substantial proportion of global deaths despite significant advances in prevention and treatment [1]. In parallel, the prevalence of metabolic disorders has increased dramatically over recent decades, with diabetes mellitus and chronic liver disease, particularly metabolic dysfunction–associated steatotic liver disease (MASLD), emerging as major public health challenges [2]. These conditions frequently coexist and share key pathophysiological mechanisms, including insulin resistance, chronic low-grade inflammation, and endothelial dysfunction. Together, these processes amplify cardiovascular risk beyond that associated with each disease alone. As a result, patients with combined metabolic and hepatic disorders represent a growing and clinically challenging population. Their cardiovascular risk is often underestimated, their vascular phenotypes heterogeneous, and traditional organ-centered models of care frequently inadequate [3]. Unmet clinical needs include the lack of integrated risk stratification strategies, limited evidence on the cardiovascular impact of targeting liver disease, and insufficient multidisciplinary approaches to prevention and management. Addressing these gaps represents a key objective of this Special Issue.

## 2. Overview of the Special Issue 

Rather than addressing isolated aspects of cardiometabolic disease, the contributions in this Special Issue form a coherent narrative that spans shared pathophysiological mechanisms, translational risk stratification, and preventive strategies in patients with diabetes and chronic liver disease. The included studies collectively illustrate how hepatic dysfunction acts as a modifier of cardiovascular risk and how this interaction can be translated into clinically meaningful markers and interventions. An overview of the published contributions and their clinical implications is summarized in Table 1 (Ref. [4, 5, 6, 7]).

**Table 1.** **Overview of the articles included in the Special Issue and their clinical implications**.

Article	Main focus	Patient population	Key message	Clinical implication
Caturano *et al*. [4]	Narrative review on diabetes, MASLD, and cardiovascular disease	Patients with type 2 diabetes and metabolic liver disease	Diabetes and MASLD act synergistically to accelerate vascular aging and worsen cardiovascular prognosis	Cardiovascular prevention and management should adopt an integrated, patient-centered approach
Mai *et al*. [5]	Original research on Fatty Liver Index and lower limb arterial calcification	Patients with type 2 diabetes	Liver steatosis indices are associated with peripheral vascular alterations	Non-invasive liver markers may contribute to cardiovascular risk assessment
Bo *et al*. [6]	Original research on systemic immune–inflammation index and outcomes after revascularization	Patients with unstable angina and diabetes	Elevated inflammatory burden predicts long-term adverse cardiovascular and cerebrovascular events	Inflammatory indices may improve prognostic stratification after coronary intervention
Li *et al*. [7]	Systematic review and meta-analysis on n-3 polyunsaturated fatty acid supplementation	Patients with type 2 diabetes	n-3 polyunsaturated fatty acid (PUFA) modestly improve lipid and glycemic indices, with heterogeneous cardiovascular effects	Nutritional strategies may serve as adjunctive tools in cardiometabolic risk reduction

MASLD, metabolic dysfunction–associated steatotic liver disease.

## 3. Conceptual Framework: Shared Cardiometabolic Mechanisms

A central theme emerging from this Special Issue is the recognition of diabetes and MASLD as components of a broader cardiometabolic continuum. Insulin resistance represents the common upstream disturbance linking these conditions. Persistent insulin resistance promotes chronic low-grade inflammation, oxidative stress, and endothelial dysfunction, all of which contribute to accelerated atherosclerosis and adverse cardiovascular remodeling [3]. In this context, the liver should no longer be viewed solely as a metabolic organ. Increasing evidence supports its role as an active driver of systemic cardiovascular risk. In fact, hepatic steatosis and dysfunction influence lipid handling, inflammatory signaling, and vascular homeostasis, thereby modulating cardiovascular outcomes [3, 8]. The narrative review included in this Special Issue provides the conceptual backbone for this framework, highlighting underappreciated contributors such as adipose tissue dysfunction and gut-derived metabolites [4]. These insights argue for moving beyond compartmentalized disease models and to consider liver disease as a clinically meaningful cardiovascular risk modifier in patients with diabetes.

## 4. Translational Evidence: Risk Stratification and Vascular Damage

Original research contributions in this Special Issue translate these mechanistic insights into clinically relevant tools for risk stratification. One study explores the relationship between the Fatty Liver Index and lower limb arterial calcification in patients with type 2 diabetes, addressing the link between hepatic steatosis and subclinical vascular damage. The association is complex, but the signal is consistent. Liver-derived indices appear to reflect broader metabolic alterations with potential vascular relevance. From a clinical perspective, such non-invasive markers may complement traditional cardiovascular risk assessment in patients with diabetes [5].

Inflammation-based risk prediction represents another important translational dimension highlighted in this Special Issue. The study evaluating the systemic immune–inflammation index in diabetic patients with unstable angina undergoing revascularization demonstrates that an elevated inflammatory burden is independently associated with worse long-term outcomes. Notably, this composite index integrates routinely available laboratory parameters, enhancing its practical applicability [6]. Inflammation-oriented risk stratification may help identify vulnerable patients beyond conventional clinical variables, supporting individualized follow-up strategies.

## 5. Therapeutic and Preventive Perspectives

Beyond risk stratification, cardiovascular risk reduction in patients with diabetes and chronic liver disease relies on a multifaceted therapeutic approach, in which lifestyle modification and established pharmacological therapies form the foundation of care [3]. Within this context, nutritional and nutraceutical interventions may provide complementary benefits, particularly in the early stages of cardiometabolic dysfunction or in patients with residual risk. Among these strategies, n-3 polyunsaturated fatty acids have attracted sustained clinical interest over time. Their role is examined in this Special Issue through a dedicated systematic review and meta-analysis. The included analysis reports modest but significant improvements in lipid and glycemic indices among patients with type 2 diabetes, while also underscoring variability in cardiovascular effects across clinical settings. These findings support a nuanced view of nutraceutical use, emphasizing personalization rather than uniform prescription [7]. Within cardiometabolic prevention, such approaches may contribute to risk reduction when integrated into comprehensive lifestyle and medical strategies [9].

## 6. Clinical Implications: Toward Integrated Care Models

The collective evidence presented in this Special Issue reinforces the need to reconsider traditional, organ-centered models of care. Cardiovascular risk in patients with diabetes and liver disease is systemic in nature, shaped by metabolic, inflammatory, and vascular interactions that transcend disciplinary boundaries [10]. As such, effective management requires multidisciplinary collaboration involving cardiology, diabetology, hepatology, and nutritional expertise. In practical terms, such integration may include shared cardiometabolic risk assessment incorporating hepatic markers, coordinated referral pathways between specialties for patients with high-risk phenotypes, and structured involvement of nutrition professionals within cardiometabolic clinics. Early identification of high-risk phenotypes may enable more timely and tailored interventions [11]. Rather than proposing rigid algorithms, the contributions point toward a pragmatic, patient-centered approach, one that adapts preventive and therapeutic strategies to the complexity encountered in everyday cardiometabolic care.

## 7. Future Directions and Unmet Needs

Despite growing awareness of the cardiometabolic continuum, several knowledge gaps remain. Longitudinal studies are needed to clarify the temporal relationships between liver disease progression and cardiovascular events, while interventional trials should assess whether targeting hepatic steatosis or systemic inflammation translates into improved cardiovascular outcomes. Further mechanistic insights are also required to better define the pathways linking metabolic dysfunction to vascular injury. In this evolving landscape, the integration of biomarkers, advanced imaging, and precision medicine approaches holds promise for refining risk stratification and guiding individualized management in complex patient populations.

## 8. Closing Remarks

In conclusion, this Special Issue provides a comprehensive and timely overview of cardiovascular outcomes and disease management in patients with diabetes and chronic liver disease. By integrating mechanistic insights with translational and preventive perspectives, the contributions underscore the clinical relevance of a more unified cardiometabolic approach. We hope that this body of work will inform clinical practice, stimulate further research, and foster interdisciplinary collaboration aimed at improving long-term cardiovascular outcomes in this increasingly prevalent and challenging patient population.
